# Exploring the information needs of patients with osteoarthritis of the knee: a content analysis of Facebook group posts

**DOI:** 10.1186/s12891-024-07240-4

**Published:** 2024-02-09

**Authors:** Nicole Schemmel, Lydia Ulrich, Julia Lauberger, Anke Steckelberg, Julia Lühnen

**Affiliations:** 1 Institute for Health and Nursing Science, Martin Luther University Halle-Wittenberg, Magdeburger Straße 8, D-06112 Halle (Saale), Germany; 2 Charité – Universitätsmedizin Berlin, corporate member of Freie Universität Berlin and Humboldt, Institute of Clinical Nursing Science, Universität zu Berlin, Berlin, Germany

**Keywords:** Information needs, Health information, Osteoarthritis of the knee, Informed decision, Total knee arthroplasty, Social media, Facebook analysis, Content analysis

## Abstract

**Background:**

End-stage osteoarthritis of the knee (OAK) is often treated by total knee arthroplasty (TKA). This intervention can significantly improve quality of life. However, many patients are dissatisfied with the outcome of surgery. One of the factors related to dissatisfaction is the of lack integration of patients’ preferences, habits and values that are not addressed by physicians. To develop realistic expectations, affected patients need evidence-based information. Our aim was to explore the information needs of patients with OAK to support the development of decision aids and consent forms to promote informed decision-making. Additionally, we investigated whether the information needs during the Covid-19 pandemic differ from those before the pandemic.

**Methods:**

The qualitative research design included a social media analysis of Facebook groups. Facebook groups were selected according to the following criteria: Thematic relevance, English or German language, at least one new post per week, from period before and after the start of the Covid-19 pandemic in March 2020. Thematically relevant group posts were analysed according to the content-structuring content analysis of Kuckartz using MaxQDA.

**Results:**

Out of 448 identified Facebook groups, we screened seven for relevant posts and a total of 77 posts out of 6 groups were selected. The following eight categories were derived during the coding process: access to health care, disease information, TKA indication and contraindication, TKA outcome and quality of life, information needs regarding conservative therapy, strain, attitude towards TKA and attitude towards conservative therapy. The analysis showed that patients with OAK need information about the benefits and risks of TKA and conservative therapies.

**Conclusion:**

This study provides information on the information needs of patients with OAK in order to decide between TKA or conservative therapy. Patients need information about treatment options in due consideration of their immediate living situation to be reliably able to assess potential outcomes. Such Information about TKA should enable patients to assess the individual prognosis with comprehensible and relevant outcome measures. Also, they should be formulated with the living environment of the patients in mind and be linked to possible fears and negative previous experiences with treatments.

**Supplementary Information:**

The online version contains supplementary material available at 10.1186/s12891-024-07240-4.

## Background

Osteoarthritis of the knee (OAK) is a degenerative musculoskeletal disorder with high relevance for the quality of life of affected persons and for the German healthcare system. A survey conducted in Germany between April 2019 and September 2020 revealed that the 12-month prevalence of OAK among women was 21.6% and among men 12.4% on average [1]. In 2021, OAK was the 15th most common main diagnosis in German hospitals with 164,536 cases [2]. The medical costs for OAK totalled 12,078 million Euros in Germany in 2020 [3]. Due to demographic change in Germany, an increase of prevalence and costs can be anticipated.

The main symptoms of OAK are pain and movement restrictions. At an advanced stage they can cause adverse effects on patients` daily life as they limit participation in relevant areas like employment, sports activities and family life [4, 5]. In 2022, Total Knee Arthroplasty (TKA) ranked 11th in the top 20 of most common surgical procedures in Germany with 199,527 cases [6]. There were 162,020 TKA interventions in the group of patients who were 60 years old and older and 37,507 TKA interventions in the age group of the under 60 years old patients [7]. TKA can improve the quality of life of affected patients. Nevertheless, surgery should be prolonged for as long as possible. Up to 95% TKAs are durable for about 10 years [5]. Because of that, younger patients are of higher risk to experience a revision surgery than older people [8]. The probability for a further joint replacement increases with every revision surgery [9]. The average age for initial implantation was 69 years in 2022 [9].

According to the guideline, TKAs are indicated when conservative therapies are exhausted and joint-preserving therapies are not an option [5, 8, 10]. A report on TKAs in Germany notes that only 43% of patients undergoing TKA are satisfied with the post-operative result [8]. Several possible reasons for dissatisfaction have been identified in different studies, including patients’ unrealistic expectations regarding the outcomes of the operation, lack of knowledge about possible complications, as well as consultations with the physicians, which focused on clinical data only while ignoring the individual situations and needs of the patients [10–13].

Exploring knowledge about information needs has the potential to get over these deficits and enables health systems, health organisations and health professionals to support informed decisions [14, 15]. Informed decisions help patients to gain autonomy and beneficence. This reflects two principles of medical ethics as discussed by Beauchamp & Childress [16, 17], as it enables patients to choose an option and to get treated according to their preferences [18]. They also meet requirements of European Charter of Patients Rights (2002), in particular the right to obtain information and the right of free choice [19].

It is necessary to provide evidence-based health information (EBHI) to enable patients to make informed decisions. Identifying information needs can serve to shape EBHI according to patients’ needs and thus also support informed shared decision-making. EBHI provides information about health decisions which is comprehensive, comprehensible, transparent, undistorted/unbiased and objective. Among other aspects they contain information about progression and effects of diseases, prevention, diagnosis, treatment, rehabilitation, aftercare and coping with the disease [20]. It is a standard for EBHI to involve the target group when preferences are determined [20]. For this study a different approach from the usual with interviews or surveys was chosen:

Searching for health information is the third most common reason for using the internet in Germany and more than half of the approximately 64 million German internet users engage in social media [21]. Social media provide a platform for different interest groups to exchange information [21–24]. Due to the popularity of social media platforms, we assumed that patients with OAK may exchange and express their information needs about possible treatment options especially in groups discussing health issues. Studies that focused on analysing and interpreting health-related content on social media platforms have been conducted before. They allowed insights into their participants demographics, aims [25, 26], characteristics of interaction between users [25], as well as their experiences with illness and treatment [27].

The current study intends to inform the development of EBHI for patients with OAK, focusing on the exploration of the information needs of these patients [28].

The research question was “What information needs do patients with OAK have with regard to the decision between surgery and conservative therapy?”

During the planning of the study, we were at the beginning of the Covid-19 pandemic. Therefore, the question arose whether the pandemic would have an impact on the information needs. We expected an additional need for information, e.g. regarding postponements of operations. Therefore, as it will be described further in the methodology section, we had to consider the time before and during the pandemic. Regarding to the development of evidence-based patient information, we wanted to ensure that this material is also useful in the future as there might be further pandemics.

This study is part of the EvAb-Pilot project which is based on the UK Medical Research Council (MRC) framework for the development and evaluation of complex interventions [29]. Our study in this research project relates to some points of the first phase of “development” of the UK MRC framework.

## Methods

The current study is a student project and was conducted from January 2021 to February 2022 by the authors – former graduate students at the Institute of Health and Nursing Science at Martin Luther University of Halle-Wittenberg. The reporting follows the Standards for Reporting Qualitative Research [30].

A systematic literature search was performed to inquire whether our research question has been explored in the past with the help of social media analysis on the Facebook platform. Inclusion and exclusion criteria were defined in advance: Publications in German and English, in which the search terms appeared in the title or abstract, were included (see Additional file 1 for the complete list of search terms). Publications dealing with a disease other than OAK (e.g. misalignments, injuries) and those published before 2004 were excluded, as Facebook was launched in 2004. The systematic literature search was accomplished between mid-January and mid-March 2021 by the two authors independently, using the literature databases Medline, PSYNDEX and PsychINFO. It did not yield any relevant results after screening titles and abstracts. To our knowledge, the question has not yet been examined using social media analysis in Facebook.

### Design

The research question was of exploratory nature and resulted in a qualitative research approach: Our aim was to explore which information patients with OAK need to decide for TKA or conservative therapy. For this purpose, Facebook posts were extracted and analysed using a qualitative content analysis according to Kuckartz [31].

### Research field and access

We chose Facebook as the most frequently used social network in the world with around 2.45 billion monthly active users [32]. Analysing Facebook groups to explore information needs of patients, we worked on a new field of research. Unlike other qualitative research methods, such as focus groups which allow interaction between researcher and participants, this was a retrospective analysis of content not specifically created for this research, so we were operating as outside observers. On the one hand, this enabled unfiltered communication without hindering participants from refraining from posing questions in front of professional healthcare providers. Additionally, the perceived anonymity in Facebook groups allow users to discuss more intimate or sensitive topics. On the other hand, it did not give us the option of specifying certain topics and clarifying questions or comments which were not easily attributed to one of our categories. However, the missing ability of an interviewer influencing on participant responses can be seen as an advantage though. Also, analysing online posts enables researchers to minimize recall bias, as current thoughts and needs are accessed [33]. We focused on Facebook groups, as these are primary places for discussion and exchange between people sharing the same interests. In contrast, profile pages serve to represent private individuals, companies, artists, etc. [34]. Consequently, groups promised to contain more intensive exchange of information concerning our topic. Facebook groups were included if they met the following inclusion criteria: They must be related to the subject of OAK or TKA, and the languages used are English or German. Open and closed groups were considered. Group activity was relevant, meaning that at least one new post should have been published per week in the particular group. This ensured that up-to-date data could be found.

To gain access to the Facebook groups for data collection, a research account was created on Facebook. We sent membership requests to the respective administrators of the groups informing them about our project. These requests contained information about the research project, data protection, and contact details of the researchers. Due to the scope of the information and the limited space in the text boxes provided for the membership request, only the most important bullet points were mentioned. However, the reference to the researchers’ profile was provided, where detailed explanations could be obtained. All the individual members of the groups were not informed personally. According to § 27 (5) of the DSAG LSA [35] this is not necessary, if the research project would otherwise not be feasible or only feasible with a disproportionate amount of effort, which was the case for our study. The creators of the Facebook posts are anonymized directly during data collection (see next section). According to § 27 (1) of the DSG LSA [35], anonymization is legitimate and demanded, as it serves to protect the creators of the posts from publication of their personal data.

### Data collection

The inclusion criteria for the group posts were based on the subject matter and were as followed: Posts covering the subject of OAK in general, posts or questions about knee TKA or conservative therapies related to OAK, posts covering the decision-making processes before a TKA, posts hinting at information missing or desired for the decision-making process, posts reviewing the decision-making processes after surgery, and finally posts about suffering from OAK. We excluded posts about other joint diseases or descriptions of a condition that cannot be directly assigned to OAK, other surgical interventions or surgical therapy descriptions that cannot be directly assigned to a TKA, decision-making processes between a TKA and another type of knee surgery, and posts about OAK in an early stage, from which no indication for a TKA will emerge in the near future. Comments were excluded unless a comment opened a new topic, then they were counted as an initial post.

We planned to include 50 posts from 2019 up to February 2020, and another 50 beginning from March 2020. With this procedure, we wanted to explore the information needs before and during the Covid-19 pandemic, because an influence of this pandemic on the information needs could not be excluded in advance. Due to technical reasons of Facebook, it is difficult to access posts that were written more than about a year ago by scrolling. For this reason, search terms need to be used that meet our inclusion criteria and thus might filter relevant posts. The following terms were used: Knie, Kniearthrose, Gonarthrose, Arthrose, Kniegelenkersatz, Kniegelenk, Prothese, Entscheidung, entscheiden, Information, unsicher, konservativ, arthrosis, knee replacement, TKA, endoprosthesis, conservative, alternative, decision, information. This approach enables to access articles from before the start of the Covid-19 pandemic in March 2020.

This study was conducted as part of a student research project with a predetermined limited time frame. As the internet, specifically Facebook, was our source for data collection, we expected a huge amount of data material that could be analysed. We assumed that we would not be able to analyse all the material to be found in the given time frame. Nevertheless, we wanted to cover the period before and during the pandemic. After consulting with a researcher, one of the co-authors, who has experience in analysing social media content, we agreed on 50 posts per time period, 100 posts in total.

The posts were viewed by the researchers and discussed regarding inclusion or exclusion while considering the criteria mentioned above. The selected group posts were copied into a Word document and the contributors were anonymized. This was done by abstracting the personal and personally identifiable data using placeholders and an anonymization list was compiled. For personal data, which may contain important information for data analysis but needed to be removed due to anonymization requirement, placeholder classes for age, occupation and place of residence were created. For example, the placeholder ‘AG1’ (age group) was inserted, when the contributor mentioned that her or his age was between 0 and 35 years old, ‘large city’ was replaced for metropolitan areas like Berlin and ‘predominantly sedentary occupation’ was replaced for occupations like programmer or other office workers. This procedure was intended to counteract the loss of content that may be important for us to understand the Facebook post in order to analyse and to determine the information needs. The document was then imported into MaxQDA, a computer software for analysing qualitative data. The data is stored in an access-protected cloud at Martin Luther University, which only members of the work group can access. They will not be passed on to external persons or institutions. The data will be deleted after a 10-year retention period [35].

### Data analysis

We performed a qualitative content analysis according to Kuckartz using MaxQDA 2020 Analytics Pro [36]. Within this method, we used the content structuring approach to summarize aspects of information needs. The steps of the structured content analysis were as followed [31, 36]:

1. After reading the Facebook posts in detail (initiating text work), memos were used to document the preliminary analysis ideas for the future categorization posts.
2. In the second phase a first coding process was made independently by two researchers in order to create preliminary main categories. The main categories were developed to structure the content. Accordingly, there were step-by-step categorizations of thematic complexes of information needs. Afterwards, the preliminary main categories were compared, discussed and determined by the two researchers. A third researcher was available for consultation in case of discrepancies. Category definitions were made and used to develop a coding guide.
3. After this, the whole data material was coded with the constituted main category system.
4. During the fourth phase, the main categories were then differentiated into subcategories. For these subcategories definitions were also consented.
5. In a repeated coding run, the text passages that had already been assigned to a main category were coded with the subcategories.
6. Summaries were created in which the Facebook posts of a subcategory were summarised thematically in a table.
7. During the last phase, results are presented and interpreted based on the main categories and subcategories with anchor examples (see results section).

A project memo was kept during the data analysis for the purpose of documenting and describing the methodical procedure and reflection [36]. We translated posts in German, which were used as anchor examples, into English and conducted a back translation. The forward translation was performed by the authors, the back translation was performed by an independent researcher.

## Results

**Fig. 1:**
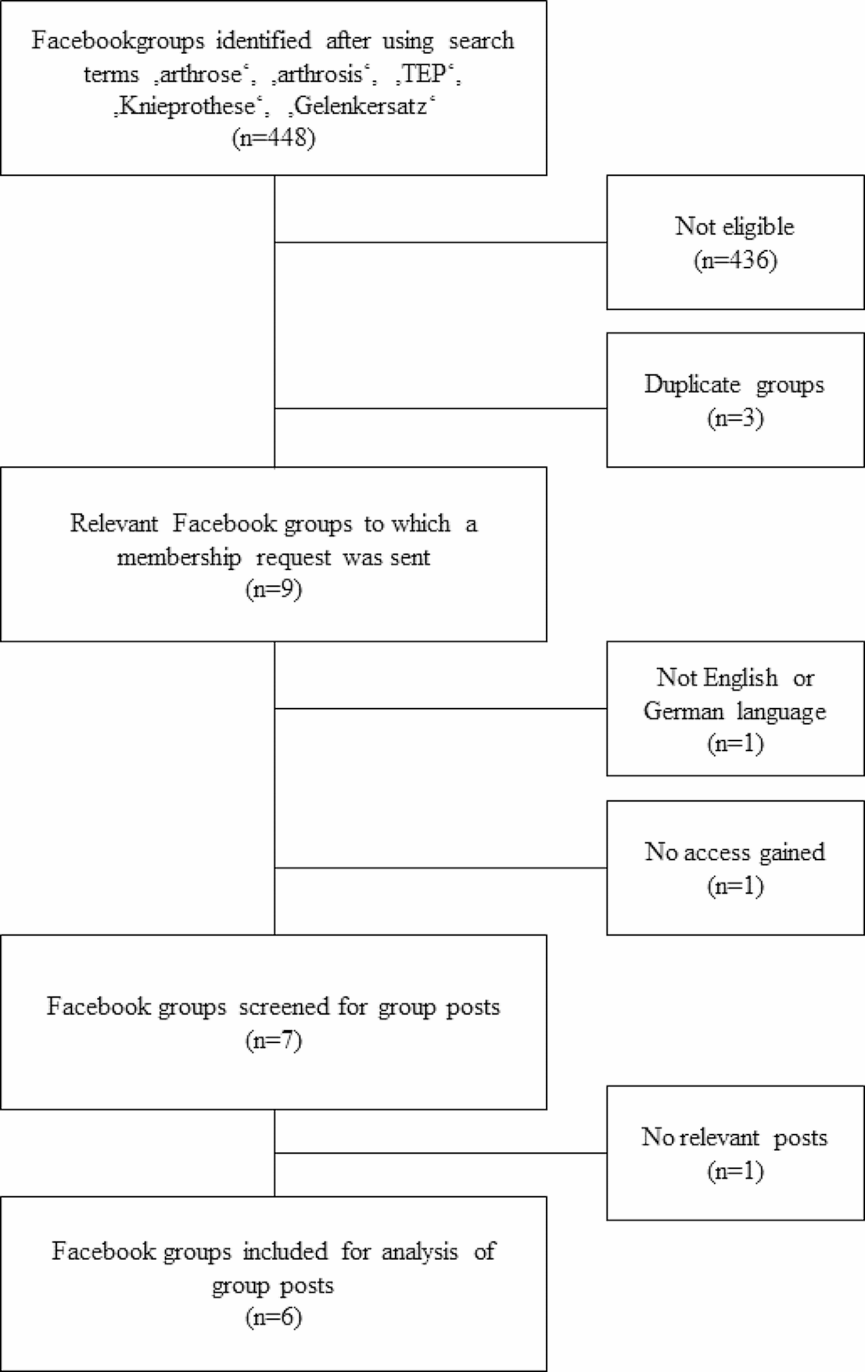
Process of including Facebook groups which were screened for relevant posts

In May 2021, we identified 448 Facebook groups of which nine were eligible for analysis considering our inclusion and exclusion criteria mentioned before. To these nine Facebook groups, membership requests were sent. We were not given access to one group resulting in an overall access to eight Facebook groups. However, one group was dropped from our analysis as it contained posts in French only, which wasn’t noticeable for us beforehand. Finally, seven Facebook groups were screened for group posts. However, of the seven groups, one group contained no relevant posts at all (Fig. 1).

We included 77 posts, 27 from the pre-pandemic period, and 50 from the pandemic period. In one group, which contained overall fewer posts, all posts could be screened by scrolling. In the remaining six groups we managed to get access to older posts by activating the filter function in the groups based on search terms as described in the methods.

Interestingly, the posts selected for our analyses were all found in German Facebook groups. No posts in English Facebook groups met the inclusion criteria. The following categories were identified: ‘Access to health care’, ‘disease information’, ‘TKA’ (with three subcategories ‘indication and contraindication’, ‘TKA outcome and quality of life’ and ‘attitude’), ‘conservative therapy’ (with two subcategories ‘information needs’ and ‘attitude’) and ‘strain’.

### Access to health care

Patients with OAK expressed information needs about the appropriate contacts to receive diagnosis and consultation, and possible ways to access them. Further, they inquired how to access outpatient treatment, hospitals, or specialized centers for the surgery, including the region in which these were located, as well as the requirements for approval of a TKA by their respective health insurance.

[P29] “[…] Can you get into the outpatient consultation hours at the university hospital […] only with a referral from a specialist doctor and an operation suggestion, or can you get into outpatient consultation hours in orthopedics without a referral […]?”

[P69] “The orthopedic surgeon – to whom I haven’t been yet – has beds in the local hospital. Now I have doubts whether it is not always better to go to a joint center? […]”.

Questions arose about the consequences of the intervention on the ability to work.

[P63] “How long did you have a sick note after the TKA surgery? Addendum: gladly also with information in which profession you work”.

### Disease information

Patients reported information needs about how to deal with the diagnosis of OAK and what it would mean for their lifestyle in the future. In this context, some contributors also inquired about the expected prognosis of the disease.

[P52] “I am overwhelmed a little bit with the diagnosis. What does it mean for me. Can I continue to pursue my profession […] still for a long time?”

Furthermore, patients questioned the investigation method that led to the diagnosis or questioned even the diagnosis itself:

[P14] “[…] For a while now I have strong pain in my hip. My attending physician says to me that my hip is o.k. and that the pain would come from the knee. A knee TKA would be the only alternative. Now I want to know from you, if you have or had similar pains or problems.”

Besides this, contributors reported their symptoms and wished to receive a reaction from fellow users to compare their disease states with those from others. Contributors also thematized the irreversibility of disease-related damage, sometimes associated with the hope of recovery.

[P76] “Can cartilage recover completely, or does it stay a trauma?”

### TKA indication and contraindication

Patients asked questions regarding the ‘right time’, indication or contraindication for TKA in connection with age, duration of illness, severity of symptoms and type of symptoms. They implied doubts about the medical justification for an indication for TKA.

[P36] “When do you know that you should have an operation? I have more or less pain in my right knee since October 2020; it’s warm, swollen […]. “.

When surgery was indicated, young age was used as an argument against surgery by both patients and treating surgeons. Young age is weighed against the severity of symptoms and an associated reduction in quality of life.

[P11] “[…] when are you “ripe” for a prosthesis? I keep hearing that I’m too young. But I want to live and run now.”

Some posts stated that from a medical point of view a TKA is unavoidable but should be delayed as long as possible. The need of information on how to delay TKA was formulated.

[P55] “I am looking for alternatives in order to delay a TKA as long as possible.”

Concomitant diseases and obesity were mentioned as factors influencing the decision-making. On the one hand, these could exacerbate disease symptoms and thus increase the urgency of TKA surgery, on the other hand, they could also increase the risk of surgery and postoperative complications.

[P65] “[…] I wanted to ask you whether there are also diabetics among you […] and whether this had an impact on the operation and aftercare, wound healing […].”

[P67] “Question to knee TKAlers: Is it true that overweight people’s knees swell more after surgery than (those of) thin people? […] Is it more difficult to bend after the operation in overweight people than in slim people?”

### TKA outcome and quality of life

The patients balanced other currently existing restrictions due to illness against expected long term improvement in quality of life. In some posts, the issue of the upcoming operation outcome was raised. Expected post-operative symptoms were assessed as problematic. The extent of the symptoms as well as their expected duration was relevant for a decision in favor of or against TKA. These two aspects were reflected in a commonly posted request for exchange of experiences and information with other contributors in these Facebook groups. Contributors expressed information needs about eventual long-term pain after surgery. They wanted to use the information to compare it with their current pain situation to inform their decision. These questions included pain-related functional limitations. Experience reports from patients who underwent the surgery were perceived as helpful when the post included the information whether surgery had sustainably alleviated pain.

[P34] “I often read here, how fine you are after knee TKA OP and that you are free of pain or sentences like: If I had known that, I would have had it done sooner. My question is: what does free of pain mean for you and what can you do again after the operation respectively where are you still limited?”

Contributors also feared a permanently existing foreign body sensation.

[P20] “I wanted to ask you how the knee feels after, is it like a foreign body? Above all I have a strange feeling about always having metal in my body then […]”.

In addition, the prospects of the occurrence of complications like loosening and iatrogenic infections were also considered relevant.

[P73] “[…] I am totally insecure. The joints last only about 2 × 15 years and then I am stuck at 70?”

Several patients expressed their desire to lead an active life in their leisure and their demand for perfect functionality in their professional lives.

[P15] “I should think about a total prosthesis. I am very active in sports. Dancing, endurance sports, weight training, as well as cycling. […] What are your experiences, how are you doing with the TKA?”

Primarily, this category was dominated by the question of whether the surgery would result in a significant and sustained improvement of their quality of life. Secondly, it included the problem of uncertainty regarding the outcome of surgery.

### Information needs regarding conservative therapy

The contributors needed an overview of possible conservative interventions as well as more specific information about the possibilities, risks and benefits of these, and the limits of their potential benefits. This applied to interventions such as oral analgesic therapy, injection therapies and various physical therapies, such as physiotherapy, appropriate sports and gymnastic exercises. Furthermore, contributors expressed insecurity concerning footwear, and benefits and side effects of other physical therapies such as ultrasound therapy. Additionally, information needs about benefits and risks of alternative medicine were expressed.

[P49] “My doctor offered me two things: STIMULATION CURRENT or ULTRASOUND. Which one do you find better? Which therapy helped you?”

[P50] “How long and how much sport do you do daily? For arthrosis and knee pain [I] don’t want to do too much […] which could be harmful, but also not do too little”.

[P62] “[…] what do I do with ACUTE PAIN? Just wait and see? Sure, lose 10 kilos, maybe orthopedic insoles in your shoes, expensive nutritional supplements, to be honest, that doesn’t really help. Does it?”

Contributors suggested or wished to reduce body weight or change their diet and needed information on that topic.

### Strain

Patients reported limited well-being up to the point of suffering. The main theme of this category is how patients deal with the reality of strain. In contrast to the category “disease information”, this one focused on the fact of strain itself. Some contributors expressed physical strain in form of pain or functional limitations.

[P2] “I don`t know how to live with the pain. I take 150 mg diclofenac per day. But nevertheless, I have bad pain.”

Patients also expressed emotional suffering in form of despair or anxiety. The fear could be directed on concomitant disease, being shocked by the diagnosis, or being overwhelmed with the need to make a decision with a perceived uncertain outcome.

[P30] “I came here in this group, because on Tuesday that I will get a knee TKA on 6.11. I am already very afraid of it, despite the long time until then. Was it the same with you, that you thought a lot about it?”

In this category, posts showed a combination of demand of information with the utterance of need of emotional support. Some posts appeared to be aimed not only at obtaining factual but also at showing empathy.

### Attitude towards TKA

In this category, the contributors expressed a basic attitude towards TKA being considered, planned, or already performed. It was influenced by expectations of treatment or the operation result. It was verbalized by emotions like uncertainty, anxiety, shock, wavering or the conviction that the intervention was going to be helpful. Some contributors showed positive or negative attitude, while others had an ambivalent view regarding the TKA.

[P10] “[…] I really have respect for this intervention.”

### Attitude towards conservative therapy

Patients expressed a basic attitude toward conservative therapies in general or regarding certain therapies. In addition, they carried out a benefit-risk-calculation resp. a cost-benefit-calculation, which was perceived as meaningful for decision-making. Some contributors seemed to be open to several forms of therapy. Therefore, they asked for general information and exchange of experiences.

[P61] “[…] want to eat healthier. Physician says it doesn`t help. But there are 1000 theories about it. There are a lot who try it out for a long time but without any success. But some make very good experiences –I too would like to. But how to begin? And what does really help?”

And still others showed a skeptical to negative attitude.

[P16] “I ask myself if conservative interventions still make sense.”

The analysis separately carried out for pre-pandemic and pandemic time period in every category revealed no difference between the periods as they were congruent for each code.

## Discussion

Our Facebook group analysis showed that information material intended for patients facing a decision for or against TKA should align with their individual understanding of their disease and individual life situation, their care goals and treatment expectations.

Our results are in accordance with previous studies which highlighted the importance of patients’ preferences for any supporting information aiding them in the decision process [10, 13, 37]. Information about TKA should aim at reducing insecurity about the outcome of the surgery by addressing indication, risks and prognosis of the procedure at a patient-appropriate level.

The result, that there was no difference between pre-pandemic and pandemic period for each code, was surprising for us. We expected an increase in the need for information and a change in topics during the COVID-19 pandemic as ‘elective’ surgeries such as hip and knee arthroplasties due to osteoarthritis were postponed to relieve stress from hospitals in Germany [38]. Sequeira et al. (2021) showed dissatisfaction with such decisions among patients [39]. Therefore, it seemed plausible that patients would express their need for sharing information and experience on how to deal with such a delay on a public forum such as Facebook. We hypothesize that a postponement of the surgery might be weighed against the risk of possible hospital-acquired Covid-19 infection, and that anxiety about that risk might outweigh the disadvantages of a postponed surgery in the patients’ eyes, and that therefore patients might refrain from engaging in such discussions.

The expectation of a successful TKA result is central to the comments of some Facebook group contributors. The single outcome expectations differ between patients and are expressed clearly. They expect to be free of pain, or at least experience significant pain reduction, they expect good mobility to live a fulfilling professional and private life. Questions asked about the ability to continue working in their professions might focus on a potential financial loss due to the long recovery phase and must be viewed as an important point of decision for or against a TKA, if only temporarily. Some patients focus on resuming certain leisure activities like dancing. Patients frequently wondered whether TKA will significantly improve their quality of life over a longer time period. This relates to the fear that the surgery could worsen the state of health. Mahdi et al. demonstrated the extents of patients’ disappointment one year after surgery due to lack of improvement [40]. One systematic review demonstrated that pre-operative expectations are an important factor for satisfaction after TKA [13]. Another systematic review highlights expectations of surgery outcomes as well as the extension of strain, especially pain and decreased functionality, as influential for decision [41]. These studies suggest that the information needs expressed by the contributors in our study are relevant and purposeful, and that decision-making processes oriented along these needs may improve satisfaction.

We therefore conclude that patient-centered information should objectively cover expectable results and enable a realistic view on the operation outcome. It is important that this information is provided in a way that is appropriate to the patient’s experience and level of education. Patients with concomitant diseases in particular inquire information about their special risks of complications.

Obesity is a risk factor for developing OAK and increases risk of surgical complications, therefore weight loss has been recommended for patients with OAK in systematic reviews and the American guideline for the management of osteoarthritis [42]. In the relevant age group of 65–75 in Germany, 49% of men resp. 36% of women, are overweight (BMI 25–30), and 22% of men resp. 19% of women are severely obese (BMI > 30) [43]. We thus assumed that questions about the influence of possible overweight on a decision regarding possible TKA would be posted in Facebook groups focused on OAK and this topic indeed occurred. But surprisingly, weight loss was not discussed as an alternative to surgery. We considered possible explanations for this. On the one hand, patients might not consider their weight as problematic and therefore do not perceive it as problem, on the other hand, we postulate that weight loss requires considerable initiative and effort by the patients, and is therefore ignored as long as possible. Although our study suggests that the information needs of patients on the influence of excess weight were low, we still suggest that motivational aspects and activating therapies are included in future materials on OAK management.

Our research indicates that patients require information not only on TKA itself, but also on conservative options. Furthermore, it should address disease progression and the health and socioeconomic burden of this disease over years. Combining information with emotional support about the strain the disease poses on quality of life and disturbances in everyday living may empower patients with OAK to reach preference-sensitive decisions in the process of shared decision-making [44].

Shared decision-making is declared as an active and collaborative exchange of information between patient and physician [45]. The consideration of pro and contra arguments is one part of the shared decision-making process [46]. In addition to Face-to-Face consultation between patients and physicians, EBHI should be made available for patients. So they can get detailed information about chances and risks of conservative therapy and of surgery and deal with it also outside of the consultations. A further part of shared decision-making is the elicitation of patients’ values and preferences [15, 46]. Preferences are to be understood as the most favored options of patients based on their attitudes respectively the specifics of every option [18].

The results of our study may inform the development of EBHI and facilitate shared decision making [14, 15, 18]. “According to Beauchamp & Childress, from a perspective of medical ethics, approaches resulting from our study might help to enhance autonomy of affected persons” [16].

As implications for care and practice in the German healthcare system, we suggest to disseminate EBHI to orthopaedic and physiotherapeutic practices and health centers. Besides orthopaedics, health care professionals like e.g. physiotherapists should be encouraged to deliver the health information to patients. EBHI may also be applied to shared decision making consultations or may be integrated into patient education programs.

### Strengths and limitations

The strengths of our study were shown by the fact that in the first step we selected and included the posts independently of each other, that we consequently excluded posts, whose authors could not clearly be identified as diagnosed with OAK at an advanced stage, that we selected posts from several Facebook groups and that we carried out the coding process independently of each other in the first step. Our study also has limitations. Instead of the planned 100 posts, we could only include 77 posts. We identified two aspects for not reaching our target: Firstly, it was difficult for us to access Facebook posts that were written more than about a year ago due to technical reasons of Facebook. Secondly, fewer posts than expected were eligible for inclusion in the respective Facebook groups. Since the contributors of the posts are supposedly medical laypersons, the topic of the posts was frequently not clearly defined from a professional point of view. Unclear cases were excluded. However, this might have influenced our analysis. In addition, the study design doesn’t allow to determine information about the contexts in which the subjects lived or were treated in. Consequently, we could not figure out, what effect different settings might have on information needs of patients. Also, the data collection was restricted to groups that used either the German or the English language, so we were unable to collect posts from all potential Facebook groups on the topic of OAK. As we unexpectedly found that we could collect German-speaking group posts only, our findings are limited to the experience of those in German-speaking healthcare systems. Germany has a dual healthcare system with a combination of statutory and private health insurance. Most of the population is covered by statutory health insurance [47]. As a result, information relating to the costs and financing of treatments covered by insurance, e.g. for TKA, might be less of interest, which may differ significantly from a population in other healthcare systems. In addition, there is the right to freely choose their physician for people in Germany, which means that they can see physicians or specialists of their choice [47]. This also includes easy access to second opinions. These facts may also differ from other healthcare systems and influence the nature of the information needs. However, the transferability regarding the development of the information sheets for the patient clientele is better in German-speaking countries. The notion that German-speaking patients appear to be more active in discussing health topics might hint at either a more open attitude towards discussing this intimate subject, or possibly a systematic lack of patient education about TKA in German-speaking countries. Further cross-cultural studies should be conducted to elaborate on this.

## Conclusions

This study provides further information on the information needs of patients with OAK to make a decision between TKA or conservative therapy. These results may guide the development process of evidence-based patient information and informed consent processes.

Patients need information about the benefits and risks of TKA as well as of conservative therapies. Patient-centered information material should enable patients to comprehensively assess the risks and prognosis of potential treatments taking the progress of the disease and personal life situations into account while empathically addressing the disease burden and possible alleviation through treatments, but also ensuring that the expectations are realistic.

Regarding future research and the aim of the EvAb-Pilot project as well as other studies aiming at providing evidence-based decision-making tools for healthcare topics, we suggest that further qualitative methods like participating observation of doctor-patient communication could be useful. In addition, these approaches could be combined with quantitative research, for instance using questionnaires to assess patients’ confidence in decision-making. This would enable healthcare providers to offer patients individually suited information, which may improve patient satisfaction.

### Electronic supplementary material

Below is the link to the electronic supplementary material.

Additional file 1

## Data Availability

The data analysed in this study are available from the corresponding author upon request.

## Abbreviations

Covid: 19 Corona Virus Disease 2019
EvAb-Pilot: Project for the development, piloting, and evaluation of evidence-based informed consent forms for total knee arthroplasty
MaxQDA: Max (Weber) Qualitative Data Analysis
OAK: Osteoarthritis of the knee
OP: Operation
TKA: Total knee arthroplasty
UK-MRC: United Kingdom Medical Research Council
