# Supplementary material for: Exploring the information needs of patients with osteoarthritis of the knee: a content analysis of Facebook group posts
Source: BMC Musculoskelet Disord. 2024 Feb 9;25:120. doi: 10.1186/s12891-024-07240-4 (PMC10854127; doi:10.1186/s12891-024-07240-4)
Supplement: Supplementary file 1 — Additional file 1 [file 12891_2024_7240_MOESM1_ESM.docx]

**Search terms for literature review**

We conducted a systematic literature search to check whether our research question 'What information needs do patients with osteoarthritis of the knee have with regard to the decision between surgery and conservative therapy? " had already been explored with the help of a social media analysis on Facebook. To identify suitable search terms, an exploratory search was carried out in the PubMed, Medline and PsychINFO literature databases at the beginning of January 2021. Translations and synonyms were determined for the terms. Search strings were then defined that were compatible with the respective databases. For this purpose, the words of a respective module were linked with the Boolean operator OR, and the modules were linked with each other using the Boolean operator AND. Truncations were also used. (see table 1)

| Table 1: Search terms | |
| --- | --- |
| Modul 1: Information needs | Modul 4: Conservative therapy |
| Aufklärung  Bedarf(e)  Bedürfnis(se)  Frage(n)  Information(en/bedürfnis/bedarf)  Hinweis(e)  question(s)  education(al) need(s)  information need(s) | Konservative Therapie(n)  conservative therap(y/ies)  Nicht-operativ  non-operativ(e)  non-surgical  orthopäd(ie/isch)  orthopaed(y/ic)  Sport(s)  exercise(s)  Physiotherap(ie/y)  physical therapy  thermotherapy(ie/y)  medical treatment(s)  medical therap(y/ies)  medication(s)  Schmerz(linderung/reduktion/therapie)  pain reduction  pain therap(y/ies)  Akupunktur  acupuncture  Eigenbluttherapie  self-blood therapy  Stammzellentherapie  stem cell therapy  injection(s)  Injektion(en)  präventi(on/v)  prevention(al)  Ernährung  nutrition |
| Modul 2 : Population of investigation |  |
| Kniegelenksarthrose  Kniearthrose  knee arthrosis  Gonarthros(e/is)  Osteoarthritis |  |
| Modul 3: field of investigation |  |
| Facebook(gruppen/groups)  Soziale Medien  Sozial(e) Netzwerk(e)  social media(s)  social network(s) |  |

| Modul 5: Surgical therapy (TEP) | Modul 6: Desicion making |
| --- | --- |
| Knie-Totalendoprothese(n)  Knie-TEP(s)  total-Gelenkendoprothe(se/thik)  Knieendoprothe(se/tik)  Knee TEP(s)  Joint replacement(s)  Total knee arthroplast(y/ies)  knee surger(y/ies)  joint surger(y/ies)  total knee replacement(s) | Decision(s)  Entscheidung(sfindung/skriterien)  Gemeinsame Entscheidung(sfindung)  common decision (making)  shared decision making  Partizipative Entscheidung(sfindung)  Informierte Entscheidung(sfindung)  informed decision (making)  decision criteri(um/a) |
